# Functional Outcome and Quality of Life After Hypoglossal-Facial Jump Nerve Suture

**DOI:** 10.3389/fsurg.2020.00011

**Published:** 2020-03-19

**Authors:** Gerd Fabian Volk, Maren Geitner, Katharina Geißler, Jovanna Thielker, Ashraf Raslan, Oliver Mothes, Christian Dobel, Orlando Guntinas-Lichius

**Affiliations:** ^1^Department of Otorhinolaryngology, Jena University Hospital, Jena, Germany; ^2^Facial Nerve Center Jena, Jena University Hospital, Jena, Germany; ^3^Department of Otorhinolaryngology, Assiut University Hospital, Assiut, Egypt; ^4^Department of Computer Science, Friedrich Schiller University, Jena, Germany

**Keywords:** facial nerve, hypoglossal nerve, facial paralysis, facial muscles, patient reported outcome measure, quality of life

## Abstract

**Background:** To evaluate the face-specific quality of life after hypoglossal-facial jump nerve suture for patients with long-term facial paralysis.

**Methods:** A single-center retrospective cohort study was performed. Forty-one adults (46% women; median age: 55 years) received a hypoglossal-facial jump nerve suture. Sunnybrook and eFACE grading was performed before surgery and at a median time of 42 months after surgery. The Facial Clinimetric Evaluation (FaCE) survey and the Facial Disability Index (FDI) were used to quantify face-specific quality of life after surgery.

**Results:** Hypoglossal-facial jump nerve suture was successful in all cases without tongue dysfunction. After surgery, the median FaCE Total score was 60 and the median FDI Total score was 76.3. Most Sunnybrook and eFACE grading subscores improved significantly after surgery. Younger age was the only consistent independent predictor for better FaCE outcome. Additional upper eyelid weight loading further improved the FaCE Eye comfort subscore. Sunnybrook grading showed a better correlation to FaCE assessment than the eFACE. Neither Sunnybrook nor eFACE grading correlated to the FDI assessment.

**Conclusion:** The hypoglossal-facial jump nerve suture is a good option for nerve transfer to reanimate the facial muscles to improve facial motor function and face-specific quality of life.

## Introduction

Facial nerve reconstruction after complex damage of the peripheral facial nerve, especially if the central stump of the facial nerve is not available for nerve suture, or after longer denervation time, still is a challenge. The hypoglossal-facial jump nerve suture (i.e., a side-to-end nerve suture of the incised hypoglossal nerve to a nerve graft that is then sutured end-to-end to the distal facial nerve) is a well-established standard procedure for facial nerve reanimation for patients with permanent facial nerve paralysis (1–4). The jump technique has overcome the until then popular classical hypoglossal-facial nerve suture with transposition of the complete hypoglossal nerve and end-to-end nerve suture directly to the facial nerve. The complete transection of the hypoglossal nerve unavoidably resulted in homolateral paralysis and hemitongue atrophy. Due to the tongue dysfunction many patients complained of permanent swallowing and speech problems (2). These problems are not seen after the jump technique with preservation of the function of the hypoglossal nerve.

Nevertheless, although hypoglossal-facial jump nerve suture and other techniques are well-established, clinical outcome research still is challenging due to a lack of standardization of outcome measurements after facial reanimation surgery (5–7). The Sunnybrook Facial Grading Scale seems to be the most robust grading scale out of the classical physician-based grading systems (8, 9). It became also popular in the last years to use the eFACE, an electronic and digitally graded facial measurement scale, as a modern alternative for physician-based facial grading (10). Further on, patient-reported outcome measures (PROMs) like the Facial Clinimetric Evaluation (FaCE) survey or the Facial Disability Index (FDI) as face-specific quality of life measures are essential to assess the facial disability in a holistic manner (11, 12).

Therefore, the present retrospective clinical study was performed to analyze systematically the outcome of hypoglossal-facial jump nerve suture using a standardized set of outcome measures including the Sunnybrook Facial Grading Scale, the eFACE, the FaCE, and the FDI.

## Materials and Methods

### Study Design and Setting

This retrospective observational study included all patients who received a hypoglossal-facial jump nerve suture at the Department of Otorhinolaryngology, Jena University Hospital, Jena, Germany, between 2007 and 2017. This period was chosen to allow a sufficient follow-up for all patients. The local institutional review board approved the study protocol for this retrospective data analysis. In general, a facial nerve reconstruction technique was chosen if electromyography (EMG) confirmed a complete denervation of facial musculature or of parts of the facial musculature. A hypoglossal-facial jump nerve suture was proposed to the patient in different scenarios: First, the procedure was indicated if an early reconstruction within 12 months after onset of the lesion was possible, but the facial nerve stump proximal to the lesion was not available, or if the proximal stump was available but the defect was that large that a combined approach was needed (3, 4, 13). Second, the procedure was indication in cases of early reconstruction within 12 months after lesion, but the lesion site was far proximal, especially if proximal to the tympanic segment of the facial nerve. Finally, the procedure was indicated if late reconstruction later than 12 months after onset of the palsy was pursued. Denervation time longer than 24 months alone was no contraindication for hypoglossal-facial jump nerve suture. Nevertheless, the patients were informed that reconstruction results later than 12 months after lesion are might be worse than in patients who underwent surgery with a denervation time of <12 months. In any case, visibility of facial musculature was confirmed by facial muscle ultrasonography. If present, almost all mimic muscles can be visualized with sonography. Details are presented elsewhere (14). Furthermore, the viability and contractibility of facial muscles was checked by direct surface electrostimulation of the muscles using a electrostimulation device (Paresestim, Krauth+Timmermann, Hamburg, Germany).

A standard hypoglossal-facial jump nerve suture technique was performed by the help of an operation microscope and 10/0 microsurgical suture material (2, 13). In all cases, the greater auricular nerve was used as interpositional nerve graft. Briefly, the hypoglossal-nerve was incised to one third. A side-to end nerve suture was performed between the incised hypoglossal nerve and the greater auricular nerve. In case of the standard hypoglossal-facial jump nerve suture, the other stump of the greater auricular nerve was sutured end-to-end to the main trunk of the peripheral extratemporal facial nerve. In cases with denervation of only parts of the peripheral facial nerve, the distal stump of the greater auricular nerve was sutured end-to-end only to the affected facial nerve branches. In a few cases of residual but functionally insufficient activity of the complete peripheral facial nerve, this distal suture to the facial nerve was performed side-to side, i.e., only the epineurium of the peripheral main trunk of the facial nerve was only incised and the distal stump of the greater auricular nerve was sutured end-to-side to the facial nerve.

The patients had follow-up visits every 3 months the first year after surgery, and every 6 months beginning with the second year. Patients' charts were reviewed for demographic characteristics, patients' history, and surgical techniques. Preoperative and postoperative evaluation included facial nerve grading using the Sunnybrook grading (8). The Sunnybrook Facial Grading Scale is a regional weighted system that rates three subscores: resting symmetry, the degree of voluntary facial muscle movement, involuntary muscle contraction (synkinesis). The three subscores are used to calculate a composite score (0 = total paralysis; 100 = normal function). An addition, the electronic facial assessment scale, eFACE, was used (15). The 16-item eFACE uses physician-graded visual analog scales and consists of 5 static, 7 dynamic, and 4 synkinesis items. The items are used to calculate static, dynamic, and synkinesis subscores, a total score, and three zonal domain scores, i.e., a periocular, midface and smile, and a lower face and neck score. Most scores range from 0 (worst) to 100 (best). Four items regarding static characteristics and items regarding dynamic characteristics relate to the nasolabial fold are rated on a scale of 0 (complete flaccidity) via 100 (balanced aesthetic appearance) to 200 (worst imaginable hypertonia). Two patient-reported outcome measures (PROMs), the Facial Clinimetric Evaluation (FaCE) scale, and the Facial Disability Index (FDI), were used (11, 12). The FaCE scores range from 0 (worst) to 100 (best). The FDI is divided into two domains and includes physical function and social/well-being function. The physical function scale is scored from −25 (worst) to 100 (best), while the social/well-being function scores from 0 (worst) to 100 (best). If several preoperative evaluations (Sunnybrook, FaCE, and FDI) were available, the evaluation closest before to surgery was selected. Out of the regular postoperative evaluations during the follow-up, the last postoperative evaluation was used.

### Statistics

All outcome variables were analyzed with IBM SPSS software for medical statistics (Version 25; IBM. New York). Data are presented as frequencies or mean ± standard deviation (SD) if not otherwise indicated. Spearman's correlation was used for univariate correlation analysis. Preoperative and postoperative Sunnybrook gradings were compared with the non-parametric Wilcoxon test for paired data. Linear regression analyses including parameters from univariate analysis and *p* < 0.01 were performed to evaluate predictors for postsurgical FaCE subscores. The significance level was set at *p* < 0.05.

## Results

### Baseline and Surgical Characteristics

Forty-one (41) patients (46% women; median age: 55 years) were included. Details on the characteristics of the patients are summarized in Supplementary Table 1. A classical hypoglossal-jump nerve suture was performed in most cases (68%). The variations of the suture technique are presented in Table 1. The median time between lesion and facial nerve reanimation surgery was 14 months (range: 0–307). All cases showed reinnervation of the facial muscles as controlled by needle EMG. A functional deficit of the tongue did not occur in any case. Thirty-three out of 41 cases (80.5%) also received an upper eyelid weight the day of hypoglossal-jump nerve suture. About half of the cases received further small surgical interventions later on during the follow-up period.

**Table 1:** Surgical characteristics of the patients (*N* = 41).

**Parameter**	**Absolute (*N*)**	**Relative (%)**
Hypoglossal-facial-jump nerve suture		
side-to-end of main trunk of facial nerve	28	68.3
side-to-side of main trunk of facial nerve	4	9.8
side-to-end of cervicofacial main branch as part of a combined approach)	5	12.2
side-to-end of cervicofacial main branch	1	2.4
side-to-end of marginal mandibular branch	2	4.8
side-to-end of zygomatic branch	1	2.4
Brow lift	6	14.6
Upper eyelid weight	33	80.5
Upper eyelid plasty	3	7.3
Lower eyelid plasty	20	48.8
Sling plasty of the corner of the mouth	3	7.3
	**Mean** **±** **SD**	**Median, range**
Denervation time, months	28.5 ± 51.2	14.0, 0–307
Time to first electrophysiological signs of reinnervation, months	5.6 ± 3.3	5.0, 2.0–14.0
Follow-up after surgery, months	39.5 ± 30.5	31.5, 6–134

*SD, standard deviation*.

### Functional Outcome and Quality of Life

The median time to the evaluation of the facial nerve function and quality of life was 42 months. The results of the Quality of life measurements using the FaCE and FDI after facial nerve reanimation with hypoglossal-facial jump nerve suture are presented in detail in Table 2. Out of the FaCE subscores, median values better than 70 points (best score: 100 points) were reached for FaCE Facial comfort (75.0 points), FaCE Oral function (75.0 points), and FaCE Social function (87.5 points). Using the FDI, more than 70 points were reached only for the FDI Social/well-being function subscore (82.0 points). The median FDI Physical function subscore was 65.0 points.

**Table 2:** Quality of life after hypoglossal-facial jump nerve suture (*N* = 41).

**Parameter**	**Mean ± SD**	**Median, range**
FaCE		
FaCE Facial movement	25.0 ± 22.0	16.6, 0–75
FaCE Facial comfort	67.3 ± 25.2	75.0,17–100
FaCE Oral function	68.52 ± 28.7	75.0,13–100
FaCE Eye comfort	40.3 ± 28.9	37.5, 0–100
FaCE Lacrimal control	52.8 ± 32.8	50.0, 0–100
FaCE Social function	75.2 ± 26.5	87.5, 6–100
FaCE Total score	56.5 ± 17.6	60.0, 23–83
FDI		
FDI Physical function	67.9 ± 17.5	65.0, 35–100
FDI Social/well-being function	75.1 ± 16.8	82.0, 40–96
FDI Total*	65.5 ± 15.5	76.3, 38–96
	**Mean** **±** **SD**	**Median, range**
Interval between surgery to postoperative evaluation, months	48.9 ± 39.6	42.0, 6–131

SD, standard deviation; *mean score of FDI Physical function and FDI Social/well-being function.

**Figure 1:**
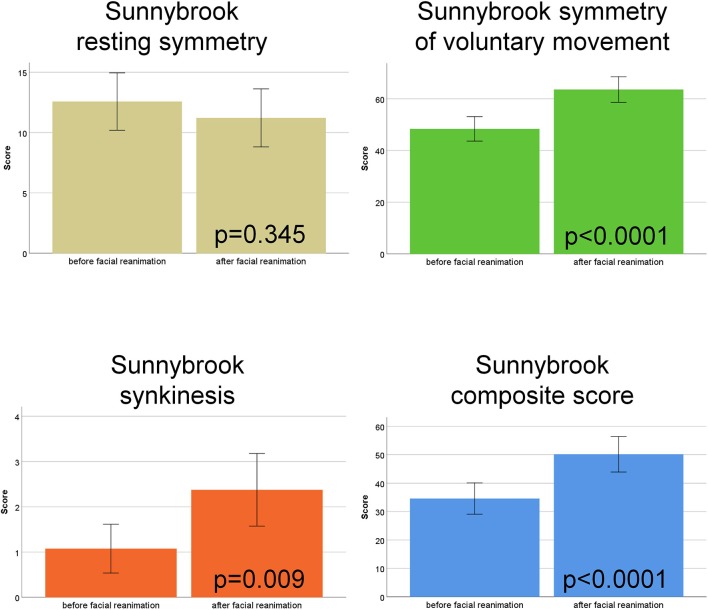
Sunnybrook facial nerve grading. Comparison of the scores before vs. after hypoglossal-facial jump nerve suture. The four subscores (Resting symmetry, Symmetry of voluntary movement, Synkinesis, Composite score) are shown in different colors.

The functional assessment with the Sunnybrook grading system showed a significant improvement for the Symmetry of voluntary movement subscore (*p* < 0.0001), a higher (worse) Synkinesis subscore (*p* = 0.009), and improvement the Composite score (*p* < 0.0001) after hypoglossal-facial jump nerve suture compared to the preoperative function. The Resting symmetry subscore was not improved (*p* = 0.345; Figure 1; Supplementary Table 2).

**Figure 2:**
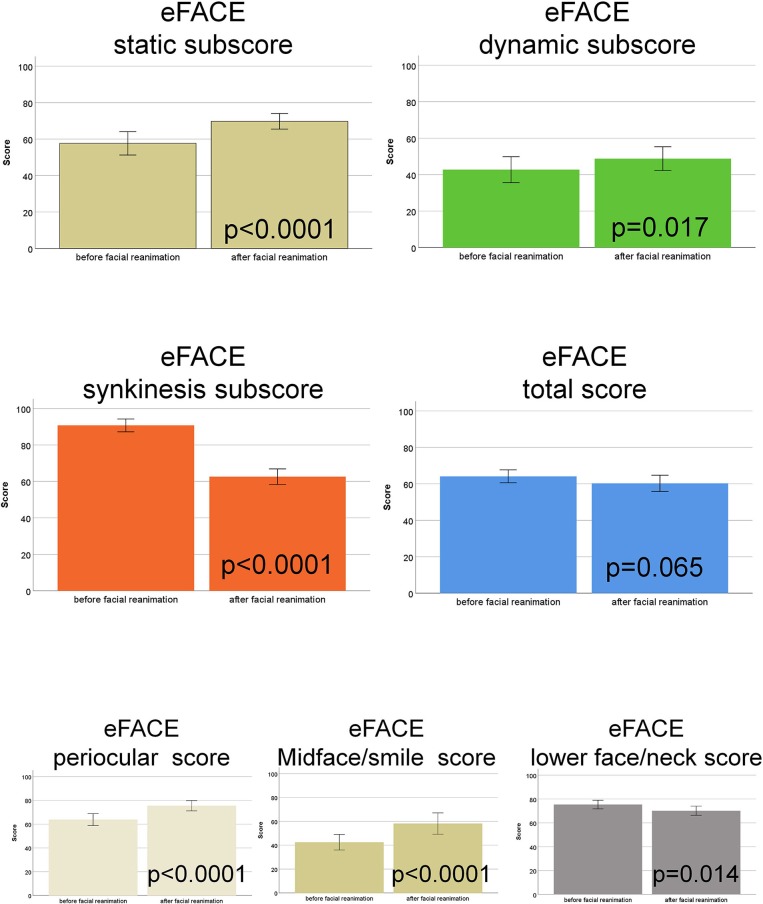
eFACE grading. Comparison of the scores before vs. after hypoglossal-facial jump nerve suture. The four subscores (static, dynamic, synkinesis, total score) and the three zonal scorers (periocular, midface/smile, lower face/neck) are shown in different colors.

The functional grading with the eFACE revealed an improvement of the Static subscore (*p* < 0.0001), the Dynamic subscore (*p* = 0.017), and a lower (worse) Synkinesis subscore (*p* < 0.0001). As a result, the eFACE total score was not significantly changed (*p* = 0.065). From the eFACE zonal scores, the Periocular score and the Midface and smile score were improved (both *p* < 0.0001), whereas the Lower face and neck score was decreased (*p* = 0.014) after facial reanimation surgery (Figure 2; Supplementary Table 3).

**Figure 3:**
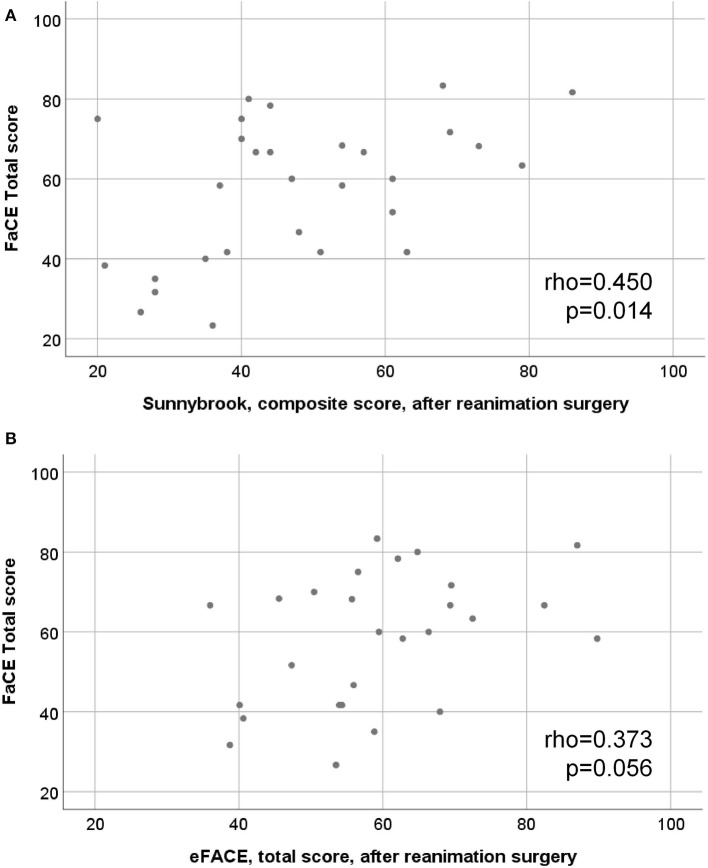
Scatter plots of the relation between motor function and quality of life assessments. **(A)** final Sunnybrook grading, composite score, after hypoglossal-facial jump nerve suture (x-axis) vs. the FaCE total score (y-axis). **(B)** final eFACE, total score grading after hypoglossal-facial jump nerve suture (x-axis) vs. the FaCE total score (y-axis).

There was a good correlation between the Sunnybrook composite score after facial reanimation and the FaCE Total score (rho = 0.450; *p* = 0.014; Figure 3). There was a moderate correlation between the eFACE Total score and the FaCE Total score after surgery (rho = 0.373; *p* = 0.056; Figure 3). The correlation between the FaCE and FDI subscores among each other is shown in Supplementary Table 4. The ratings of the FaCE subscores were more independent among each other than the FDI subscores. The correlation between the Sunnybrook and eFACE subscores among each other is shown in Supplement Table 5. Only some of the Sunnybrook and eFACE subscores correlated to each other.

The Supplementary Tables 6–9 contain the univariate correlation analyses for association between patients' und surgical characteristics and outcome in FaCE, FDI, Sunnybrook, and eFACE grading, respectively. Younger age and sometimes a higher Sunnybrook composite score were correlated to higher FaCE subscores (all *p* < 0.05). Additional upper eyelid weight implantation was associated to a higher FaCE Eye comfort subscore (*p* = 0.014). Additional upper eyelid weight loading and a higher eFACE total score was associated to a higher FDI Physical function subscore (*p* = 0.047 and *p* < 0.0001, respectively). The final Sunnybrook composite score and also the final eFACE total score did not show any associations to patients' und surgical characteristics (all *p* > 0.05). Finally, the multivariate linear logistic regression analyses are presented in Table 3. Younger age (beta = −0.388; 95% confidence interval [CI] = −0.036 to −0.739) and a higher final Sunnybrook composite score (beta = 0.479; CI = 0.053 to 0.904) were independent predictors for a higher (better) FaCE Facial movement subscore. Younger age was also an independent predictor for higher FaCE Facial comfort subscore (beta = −0.543; CI = −1.004 to −0.083), FaCE Oral function subscore (beta = −0.565; CI = −1.131 to −0.0001), FaCE Eye comfort subscore (beta = −0.754; CI = −1.215 to −0.293), FaCE Lacrimal control subscore (beta = −1.119; CI = −1.651 to −0.586), and a higher FaCE total score (beta = −0.335; CI = −0.633 to −0.038). Additionally, upper lid loading was an independent predictors for a higher FaCE Eye comfort subscore (beta = 29.038; CI = 8.995 to 49.081).

**Table 3:** Linear regression analysis for independent predictors of the FaCE and FDI subscores.

**Measure**	**beta**	**95% CI** **lower**	**95% CI** **upper**	Stand.^*^ beta	p^**^
**FaCE Facial movement;** *R*^2^ = 0.684; *p* = 0.001					
Side (right = 0; left = 1)	14.377	−0.381	29.136	0.330	0.056
Age	−0.388	−0.036	−0.739	−0.333	**0.032**
Sunnybrook, composite score, after facial reanimation surgery	0.479	0.053	0.904	0.380	**0.029**
**FaCE Facial comfort;** *R*^2^ = 0.659; *p* = 0.011					
Age	−0.543	−1.004	−0.083	−0.440	**0.023**
Interval between onset of palsy to surgery (denervation time)	0.051	−0.089	0.192	0.137	0.456
Sunnybrook, composite score. after facial reanimation surgery	0.453	−0.045	0.951	0.341	0.072
eFACE, total score, after facial reanimation surgery	0.325	−0.324	0.974	0.194	0.310
**FaCE Oral function;** *R*^2^ = 0.367; *p* = 0.050					
Age	−0.565	−1.131	0.000	−0.367	**0.050**
**FaCE Eye comfort;** *R*^2^ = 0.504; *p* < 0.0001					
Gender (female = 0; male = 1)	14.614	−2.508	31.736	0.254	0.091
Age	−0.754	−1.215	−0.293	−0.476	**0.002**
Additional upper eyelid weight (no = 0; yes = 1)	29.038	8.995	49.081	0.433	**0.006**
**FaCE Lacrimal control;** *R*^2^ = 0.646; *p* < 0.0001					
Age	−1.119	−1.651	−0.586	−0.646	** < 0.0001**
**FaCE Social function**					
Not applicable					
**FaCE total score;** *R*^2^ = 0.652; *p* = 0.013					
Side (right = 0; left = 1)	2.949	−9.754	15.652	0.088	0.635
Age	−0.335	−0.633	−0.038	−0.383	**0.029**
Sunnybrook, composite score, after facial reanimation surgery	0.303	−0.091	0.696	0.321	0.125
eFACE, total score, after facial reanimation surgery	0.364	−0.086	0.814	0.306	0.108
**FDI Physical function;** *R*^2^ = 0.409; *p* = 0.111					
Additional upper eyelid weight (no = 0; yes = 1)	14.560	−0.381	29.501	0.375	0.056
eFACE, total score, after facial reanimation surgery	0.185	−0.296	0.666	0.148	0.435
**FDI Social/well-being function**					
Not applicable					
**FDI Total score**					
Not applicable					

* Standardized beta;

** *p-values < 0.05 in bold*.

## Discussion

The hypoglossal-facial jump nerve suture is part of the standard repertoire of facial nerve repair techniques. Several case series have shown that the jump technique is safe in regard of the preservation of the tongue function and leads to satisfactory functional results (1, 2, 4, 16–18). It is important to differentiate the jump technique with one third incision and normally side-to-end suture to a nerve graft from the splitting technique. Here, the hypoglossal nerve is split along its length and the split segment is transposed for end-to-end suture to the facial nerve. The splitting technique risks more injury to hypoglossal axons and results in mild to moderate hemitongue atrophy because many interweaving axons are transected (1, 19, 20).

Nevertheless, classical assessments of quality of life or with PROMs after hypoglossal-facial jump nerve suture are sparse. Lin et al. used apart from a Sunnybrook grading also the FDI and also the short form SF-36 quality of life questionnaire to analyze several types of facial nerve repair (21). Fourteen patients with hypoglossal-facial jump nerve suture with or without interpositional graft and a Sunnybrook Composite score of 35.6–39.2 points revealed 67.1–70.1 points in the FDI Physical function subscore and 66.3–77.3 points in the Social/well-being function subscore. Due to the variability in patients' characteristics, it is difficult to compare this study with the present study, but the results FDI are in the same range, whereas functional outcome measuring with Sunnybrook grading are better in the present study. General quality of life measured with the SF-36 was highest after hypoglossal-facial jump nerve suture in the study by Lin et al. (21). General health values in the range of the normal population were reached after facial nerve repair and confirmed by another study (22). The SF-36 was not applied in the present study. It has been shown that the correlation of SF-36 and FDI or FaCE results is moderate (23, 24). Furthermore, function of individual facial regions seems not equally important for estimating facial palsy-related quality of life. The ability to smile seems to be of greatest importance (25). Therefore, one has to be careful with direct comparisons of the results of the different tools. Better facial motor function is highly correlated with better quality of life. This has also been shown by others and in general for patients with facial palsy (22, 24, 26). Furthermore, also other studies have revealed that age is negatively correlated with functional and quality of life outcome after facial nerve repair (22).

The present study is not without limitations: The retrospective design includes an uncontrolled selection bias. Although one of the largest series on hypoglossal-facial jump nerve suture, the sample size was not large. This limited the possibilities for multivariate analyses. Strengths are the standardized use of reliable functional outcome measures (Sunnybrook and eFACE) and face-specific quality of life measures (FaCE and FDI). Another advantage is the long median follow-up of 31.5 months allowing a definite estimation of the final results.

What is lacking is a universal objective tool to measure the outcome after facial nerve repair (5). We could identify only one study using objective measure to evaluate the outcome after hypoglossal-facial jump nerve suture (20). Kochhar et al. used the facial asymmetry index (FAI) and the MEEI smile and oral Facegram software (27). Kochhar et al. revealed an objective reduction of facial asymmetry and improved lip excursions for smiling after hypoglossal-facial jump nerve suture. Recently, we have introduced an automated tool based on a machine learning approach for objective facial action coding and another tool for objective grading of patients with facial palsy (28, 29). An important next task will be to apply these objective tools also for evaluation of the patients after hypoglossal-facial jump nerve suture.

## Conclusion

This retrospective cohort study on 41 patients with hypoglossal-facial jump nerve suture confirms that this cross-nerve technique is a well-deserved part of the standard armamentarium for facial nerve repair. This was confirmed not only by standard facial grading but also by PROMs allowing a better inclusion of the patient's perspective. With good patient selection, this surgery achieves a very high overall success rate. The present study demonstrates a functionally relevant improvement for facial motor function after surgery in line with a good face-specific quality of life.

## Data Availability Statement

All datasets generated for this study are included in the article/Supplementary Material.

## Ethics Statement

The studies involving human participants were reviewed and approved by the ethics committee of the Jena University Hospital.

## Author Contributions

OG-L and GV: protocol development. MG, OM, KG, JT, and AR: acquisition of the analyzed data. OG-L, GV, and CD: data analysis. All authors: critical revision of work, and final approval of the version to be published. All authors complied with all aspects of the work. They ensure that questions related to the accuracy of the work are adequately discussed and solved.

### Conflict of Interest

The authors declare that the research was conducted in the absence of any commercial or financial relationships that could be construed as a potential conflict of interest.
