# Supplementary material for: Functional Outcome and Quality of Life After Hypoglossal-Facial Jump Nerve Suture
Source: Front Surg. 2020 Mar 19;7:11. doi: 10.3389/fsurg.2020.00011 (PMC7096350; doi:10.3389/fsurg.2020.00011)
Supplement: Supplementary file 1 [file Data_Sheet_1.docx]

**supplementary tables**

**Supplementary Table 1**

| **Supplementary Table 1. Baseline characteristics of the patients (N=41)** | | |
| --- | --- | --- |
| **Parameter** | **Absolute (N)** | **Relative (%)** |
| Gender |  |  |
| Female | 19 | 46.3 |
| Male | 22 | 53.7 |
| Affected side |  |  |
| Right | 14 | 34.1 |
| Left | 27 | 65.9 |
| Etiology |  |  |
| Benign tumor | 26 | 63.4 |
| Malignant tumor | 7 | 17.1 |
| Trauma | 3 | 7.3 |
| Other | 5 | 12.2 |
| Preoperative/Postoperative paralysis* |  |  |
| Preoperative | 16 | 39.0 |
| Postoperative | 25 | 61.0 |
|  | **Mean±SD** | **Median, range** |
| Age at onset of palsy, years | 45.9±21.5 | 51, 0-76 |
| Age at reconstruction surgery, years | 48.4±19.9 | 55, 2-76 |

*Onset of the paralysis before the planned surgery for facial reanimation or paralysis occurred the day of facial reanimation surgery as the reanimation was performed as a single-stage procedure as part of cancer surgery resulting in the facial paralysis.

**Supplementary Table 2**

| **Supplementary Table 2. Sunnybrook Facial Grading System before versus after* hypoglossal-facial jump nerve suture (N=41)** | | | | | |
| --- | --- | --- | --- | --- | --- |
|  | **Before surgery** | | **After surgery** | |  |
| **Parameter** | **Mean** | **SD** | **Mean** | **SD** | **p**** |
| Resting symmetry | 12.63 | 7.05 | 11.38 | 6.98 | 0.345 |
| Symmetry of voluntary movement | 49.68 | 16.31 | 63.20 | 14.36 | **<0.0001** |
| Synkinesis | 1.05 | 1.67 | 2.38 | 2.51 | **0.009** |
| Composite score | 35.92 | 18.22 | 49.45 | 18.19 | **<0.0001** |

*last follow-up; **p-values <0.05 in bold.

**Supplementary Table 3**

| **Supplementary Table 3. eFACE grading before versus after* hypoglossal-facial jump nerve suture (N=41)** | | | | | |
| --- | --- | --- | --- | --- | --- |
|  | **Before surgery** | | **After surgery** | |  |
| **Parameter** | **Mean** | **SD** | **Mean** | **SD** | **p**** |
| **eFACE subscores** |  |  |  |  |  |
| Resting brow height | 86.82 | 25.61 | 93.75 | 18.45 | **0.041** |
| Resting palpebral fissure width | 82.66 | 50.10 | 97.30 | 38.87 | 0.068 |
| Oral commissure position at rest | 71.74 | 41.51 | 85.50 | 36.06 | **0.037** |
| Nasolabial fold depth at rest | 51.24 | 37.60 | 51.68 | 36.55 | 0.561 |
| Nasolabial fold orientation at rest | 82.68 | 34.31 | 89.28 | 32.24 | 0.137 |
| Static subscore | 57.64 | 19.54 | 63.97 | 10.53 | **<0.0001** |
| Brow elevation | 21.58 | 35.15 | 10.38 | 19.90 | 0.171 |
| Gentle eye closure | 64.18 | 34.32 | 79.53 | 26.65 | **0.044** |
| Full eye closure | 72.00 | 30.08 | 85.50 | 23.10 | **0.040** |
| Oral commissure movement | 18.53 | 25.85 | 24.15 | 19.68 | **0.014** |
| Nasolabial fold depth with smile | 29.84 | 33.54 | 39.70 | 43.39 | 0.301 |
| Nasolabial fold orientation with smile | 58.58 | 43.37 | 63.88 | 46.68 | 0.282 |
| Lower lip movement | 45.26 | 22.95 | 42.70 | 22.36 | 0.652 |
| Dynamic subscore | 43.30 | 21.30 | 48.78 | 19.49 | **0.017** |
| Ocular synkinesis | 88.87 | 18.35 | 86.55 | 21.23 | 0.639 |
| Midfacial synkinesis | 91.87 | 19.01 | 83.43 | 23.49 | 0.060 |
| Mentalis dimpling | 87.16 | 16.41 | 77.25 | 21.17 | **0.0001** |
| Platysmal synkinesis | 91.24 | 21.15 | 89.98 | 16.28 | 0.437 |
| Synkinesis subscore | 90.97 | 10.52 | 59.00 | 11.04 | **<0.0001** |
| **eFACE total score** | 63.97 | 10.53 | 57.30 | 11.94 | 0.065 |
| **eFACE zonal scores** |  |  |  |  |  |
| Periocular score | 63.19 | 15.01 | 75.50 | 12.42 | **<0.0001** |
| Midface and smile score | 42.62 | 19.29 | 59.03 | 26.37 | **<0.0001** |
| Lower face and neck score | 75.43 | 10.85 | 69.98 | 11.62 | **0.014** |

*last follow-up; ****** p-values <0.05 in bold

**Supplementary Table 4**

| **Supplementary Table 4. Correlation between FaCE and FDI subscores** | | | | | | | | | | |
| --- | --- | --- | --- | --- | --- | --- | --- | --- | --- | --- |
|  | FaCE Facial movement | FaCE Facial comfort | FaCE  Oral function | FaCE  Eye comfort | FaCE Lacrimal control | FaCE Social function | FaCE  Total score | FDI Physical function | FDI Social/well-being function | FDI Total* |
| FaCE Facial movement | - | r=-0.108;  p=0.577 | r=0.191;  p=0.322 | r=0.204;  p=0.288 | r=-0.208  p<0.289 | r=0.219;  p=0.262 | r=0.455;  p=0.013 | r=0.274;  p=0.150 | r=-0.030;  p=0.881 | r=0.172;  p=0.381 |
| FaCE Facial comfort | r=-0.108;  p=0.577 | - | r=0.413;  p=0.026 | r=0.444;  p<0.016 | r=0.526;  p=0.004 | r=0.251;  p=0.198 | r=0.530;  p=0.003 | r=0.375;  p<0.045 | r=0.472;  p<0.011 | r=0.462;  p<0.013 |
| FaCE Oral function | r=0.191;  p=0.322 | r=0.413;  p=0.026 | - | r=0.476;  p=0.009 | r=0.564;  p=0.002 | r=0.516;  p=0.005 | r=0.806;  p<0.0001 | r=0.777;  p<0.0001 | r=0.567;  p=0.002 | r=0.731;  p<0.0001 |
| FaCE Eye comfort | r=0.204;  p=0.288 | r=0.444;  p<0.016 | r=0.476;  p=0.009 | - | r=0.539  p=0.003 | r=0.302;  p=0.119 | r=0.686;  p<0.0001 | r=0.549;  p=0.002 | r=0.287  p=0.139 | r=0.542;  p=0.003 |
| FaCE Lacrimal control | r=-0.208;  p<0.289 | r=0.526;  p=0.004 | r=0.564;  p=0.002 | r=0.539  p=0.003 | - | r=0.375  p=0.054 | r=0.565;  p=0.002 | r=0.469  p=0.012 | r=0.428  p=0.026 | r=0.462  p=0.015 |
| FaCE Social function | r=0.219;  p=0.262 | r=0.251;  p=0.198 | r=0.516;  p=0.005 | r=0.302;  p=0.119 | r=0.375  p=0.054 | - | r=0.565;  p=0.002 | r=0.591;  p=0.0001 | r=0.712;  p<0.0001 | r=0.706;  p<0.0001 |
| FaCE Total score | r=0.455;  p=0.013 | r=0.530;  p=0.003 | r=0.806;  p<0.0001 | r=0.686;  p<0.0001 | r=0.565;  p=0.002 | r=0.565;  p=0.002 | - | r=0.811;  p<0.0001 | r=0.656;  p<0.0001 | r=0.656;  p<0.0001 |
| FDI Physical function | r=0.274;  p=0.150 | r=0.375;  p<0.045 | r=0.777;  p<0.0001 | r=0.549;  p=0.002 | r=0.469  p=0.012 | r=0.591;  p=0.001 | r=0.811;  p<0.0001 | - | r=0.617;  p<0.0001 | r=0.919;  p<0.001 |
| FDI Social/well-being function | r=-0.030;  p=0.881 | r=0.472;  p<0.011 | r=0.567;  p=0.002 | r=0.287  p=0.139 | r=0.428  p=0.026 | r=0.712;  p<0.0001 | r=0.656;  p<0.0001 | r=0.617;  p<0.0001 | - | r=0.844;  p<0.0001 |
| FDI Total* | r=0.172;  p=0.381 | r=0.462;  p<0.013 | r=0.731;  p<0.0001 | r=0.542;  p=0.003 | r=0.462  p=0.015 | r=0.706;  p<0.0001 | r=0.656;  p<0.0001 | r=0.919;  p<0.001 | r=0.844;  p<0.0001 | - |

*mean score of FDI Physical function and FDI Social/well-being function; Bonferroni correction revealed a p-value threshold of p = 0.05/100 = 0.0005. Significant values are highlighted in gray.

**Supplementary Table 5**

| **Supplementary Table 5. Correlation between Sunnybrook and eFACE subscores** | | | | | | | | | | | |
| --- | --- | --- | --- | --- | --- | --- | --- | --- | --- | --- | --- |
|  | SB  Resting symmetry | SB  Symmetry voluntary movement | SB  Synkinesis | SB  Composite score | eFACE  Static subscore | eFACE Dynamic subscore | eFACE Synkinesis subscore | eFACE Total score | eFACE Periocular score | eFACE Midface and smile score | eFACE Lower face and neck score |
| SB  Resting symmetry | - | r=-0.450;  p=0.004 | r=-0.338;  p=0.033 | r=-0.699;  p<0.0001 | r=-0.277;  p=0.097 | r=-0.588;  p<0.0001 | r=-0.509;  p=0.001 | r=-0.544;  p<0.0001 | r=-0.272;  p=0.089 | r=-0.735;  p<0.0001 | r=0.031;  p=0.849 |
| SB  Symmetry voluntary movement | r=-0.450;  p=0.004 | - | r=0.318;  p=0.045 | r=0.933;  p<0.0001 | r=0.043;  p=0.798 | r=0.470;  p=0.002 | r=0.241;  p=0.151 | r=0.308;  p=0.064 | r=0.186;  p=0.252 | r=0.387;  p=0.014 | r=-0.095;  p=0.562 |
| SB  Synkinesis | r=-0.338;  p=0.033 | r=0.318;  p=0.045 | - | r=0.260;  p=0.106 | r=-0.082;  p=0.631 | r=0.351;  p=0.206 | r=0.082;  p=0.628 | r=0.139;  p=0.413 | r=0.063;  p=0.700 | r=0.339;  p=0.032 | r=-0.362;  p=0.022 |
| SB  Composite score | r=-0.699;  p<0.0001 | r=0.933;  p<0.0001 | r=0.260;  p=0.106 | - | r=0.129;  p=0.447 | r=0.555  p<0.0001 | r=0.367;  p=0.026 | r=0.432;  p=0.008 | r=0.238;  p=0.140 | r=0.533;  p<0.0001 | r=-0.056;  p=0.733 |
| eFACE  Static subscore | r=-0.277;  p=0.097 | r=0.043;  p=0.798 | r=-0.082;  p=0.631 | r=0.129;  p=0.447 | - | r=0.354;  p=0.032 | r=0.883;  p<0.0001 | r=0.798;  p<0.0001 | r=0.696  p<0.0001 | r=0.359;  p=0.029 | r=0.175;  p=0.301 |
| eFACE  Dynamic subscore | r=-0.588;  p<0.0001 | r=0.470;  p=0.002 | r=0.351;  p=0.206 | r=0.555;  p<0.0001 | r=0.354;  p=0.032 | - | r=0.722;  p<0.0001 | r=0.825;  p<0.0001 | r=0.404;  p=0.010 | r=0.897;  p<0.0001 | r=0.226;  p=0.161 |
| eFACE  Synkinesis subscore | r=-0.509;  p=0.001 | r=0.241;  p=0.151 | r=0.082;  p=0.628 | r=0.367;  p=0.026 | r=0.883;  p<0.0001 | r=0.722;  p<0.0001 | - | r=0.982;  p<0.0001 | r=0.452;  p=0.005 | r=0.684;  p<0.0001 | r=0.218;  p=0.195 |
| eFACE total score | r=-0.544;  p<0.0001 | r=0.308;  p=0.064 | r=0.139;  p=0.413 | r=0.432;  p=0.008 | r=0.798;  p<0.0001 | r=0.825;  p<0.0001 | r=0.982;  p<0.0001 | - | r=0.463;  p=0.004 | r=0.765;  p<0.0001 | r=0.224;  p=0.182 |
| eFACE  Periocular score | r=-0.272;  p=0.089 | r=0.186;  p=0.252 | r=0.063;  p=0.700 | r=0.238;  p=0.140 | r=0.696  p<0.0001 | r=0.404;  p=0.010 | r=0.452;  p=0.005 | r=0.463;  p=0.004 | - | r=0.304;  p=0.056 | r=-0.020;  p=0.902 |
| eFACE  Midface and smile score | r=-0.735;  p<0.0001 | r=0.387;  p=0.014 | r=0.339;  p=0.032 | r=0.533;  p<0.0001 | r=0.359;  p=0.029 | r=0.897;  p<0.0001 | r=0.684;  p<0.0001 | r=0.765;  p<0.0001 | r=0.304;  p=0.056 | - | r=0.214;  p=0.184 |
| eFACE  Lower face and neck score | r=0.031;  p=0.849 | r=-0.095;  p=0.562 | r=-0.362;  p=0.022 | r=-0.056;  p=0.733 | r=0.175;  p=0.301 | r=0.226;  p=0.161 | r=0.218;  p=0.195 | r=0.224;  p=0.182 | r=-0.020;  p=0.902 | r=0.214;  p=0.184 | - |

Bonferroni correction revealed a p-value threshold of p = 0.05/121 = 0.00041322. Significant values are highlighted in gray.

**Supplementary Table 6**

| **Supplementary Table 6. Univariate analysis of associations to FaCE score** | | |
| --- | --- | --- |
| **Parameter** | **rho** | **p*** |
| **FaCE Facial movement** |  |  |
| Gender (female = 0. male = 1) | -0.121 | 0.532 |
| Side (right = 0; left = 1) | **0.480** | **0.008** |
| Onset (Preoperative = 0; Postoperative =1) | -0.266 | 0.162 |
| Age | **-0.318** | **0.093** |
| Malignant etiology (no = 0; yes = 1) | 0.075 | 0.697 |
| Interval onset of palsy to surgery (denervation time) | -0.198 | 0.304 |
| Interval surgery to evaluation | 0.190 | 0.324 |
| Classical hypoglossal-facial jump nerve suture (no = 0; yes = 1) | 0.023 | 0.906 |
| Additional upper eyelid weight (no = 0; yes = 1) | 0.277 | 0.146 |
| Sunnybrook, composite score, before facial reanimation surgery | **0.378** | **0.052** |
| Sunnybrook, composite score, after facial reanimation surgery | **0.538** | **0.003** |
| eFACE, total score, before facial reanimation surgery | -0.102 | 0.614 |
| eFACE, total score, after facial reanimation surgery | 0.356 | 0.068 |
| **FaCE Facial comfort** |  |  |
| Gender (female = 0. male = 1) | 0.205 | 0.897 |
| Side (right = 0; left = 1) | 0.176 | 0.361 |
| Onset (Preoperative = 0; Postoperative =1) | -0.203 | 0.291 |
| Age | **-0.469** | **0.010** |
| Malignant etiology (no = 0; yes = 1) | -0.082 | 0.672 |
| Interval onset of palsy to surgery (denervation time) | **0.340** | **0.071** |
| Interval surgery to evaluation | 0.171 | 0.375 |
| Classical hypoglossal-facial jump nerve suture (no = 0; yes = 1) | 0 | 1.000 |
| Additional upper eyelid weight (no = 0; yes = 1) | 0.117 | 0.544 |
| Sunnybrook, composite score, before facial reanimation surgery | 0.277 | 0.162 |
| Sunnybrook, composite score, after facial reanimation surgery | **0.361** | **0.054** |
| eFACE, total score, before facial reanimation surgery | **0.348** | **0.076** |
| eFACE, total score, after facial reanimation surgery | **0.379** | **0.051** |
| **FaCE Oral function** |  |  |
| Gender (female = 0. male = 1) | -0.013 | 0.948 |
| Side (right = 0; left = 1) | 0.273 | 0.151 |
| Onset (Preoperative = 0; Postoperative =1) | 0.032 | 0.870 |
| Age | **-0.470** | **0.010** |
| Malignant etiology (no = 0; yes = 1) | -0.097 | 0.618 |
| Interval onset of palsy to surgery (denervation time) | 0.181 | 0.348 |
| Interval surgery to evaluation | 0.137 | 0.480 |
| Classical hypoglossal-facial jump nerve suture (no = 0; yes = 1) | 0.025 | 0.899 |
| Additional upper eyelid weight (no = 0; yes = 1) | 0.193 | 0.317 |
| Sunnybrook, composite score, before facial reanimation surgery | 0.313 | 0.112 |
| Sunnybrook, composite score, after facial reanimation surgery | 0.311 | 0.101 |
| eFACE, total score, before facial reanimation surgery | 0.122 | 0.544 |
| eFACE, total score, after facial reanimation surgery | 0.291 | 0.141 |
| **FaCE Eye comfort** |  |  |
| Gender (female = 0. male = 1) | **0.387** | **0.038** |
| Side (right = 0; left = 1) | 0.248 | 0.194 |
| Onset (Preoperative = 0; Postoperative =1) | -0.076 | 0.694 |
| Age | **-0.408** | **0.028** |
| Malignant etiology (no = 0; yes = 1) | -0.020 | 0.916 |
| Interval onset of palsy to surgery (denervation time) | 0.174 | 0.367 |
| Interval surgery to evaluation | 0.305 | 0.108 |
| Classical hypoglossal-facial jump nerve suture (no = 0; yes = 1) | -0.078 | 0.698 |
| Additional upper eyelid weight (no = 0; yes = 1) | **0.452** | **0.014** |
| Sunnybrook, composite score, before facial reanimation surgery | 0.227 | 0.255 |
| Sunnybrook, composite score, after facial reanimation surgery | 0.076 | 0.696 |
| eFACE, total score, before facial reanimation surgery | 0.066 | 0.742 |
| eFACE, total score, after facial reanimation surgery | 0.182 | 0.364 |
| **FaCE Lacrimal control** |  |  |
| Gender (female = 0. male = 1) | 0.085 | 0.668 |
| Side (right = 0; left = 1) | 0.277 | 0.154 |
| Onset (Preoperative = 0; Postoperative =1) | 0.143 | 0.468 |
| Age | **-0.663** | **<0.0001** |
| Malignant etiology (no = 0; yes = 1) | -0.143 | 0.497 |
| Interval onset of palsy to surgery (denervation time) | 0.351 | 0.067 |
| Interval surgery to evaluation | -0.009 | 0.965 |
| Classical hypoglossal-facial jump nerve suture (no = 0; yes = 1) | 0.133 | 0.500 |
| Additional upper eyelid weight (no = 0; yes = 1) | 0 | 1.000 |
| Sunnybrook, composite score, before facial reanimation surgery | 0.067 | 0.739 |
| Sunnybrook, composite score, after facial reanimation surgery | 0.059 | 0.764 |
| eFACE, total score, before facial reanimation surgery | -0.014 | 0.945 |
| eFACE, total score, after facial reanimation surgery | 0.108 | 0.593 |
| **FaCE Social function** |  |  |
| Gender (female = 0. male = 1) | 0.096 | 0.628 |
| Side (right = 0; left = 1) | 0.163 | 0.406 |
| Onset (Preoperative = 0; Postoperative =1) | -0.038 | 0.851 |
| Age | -0.184 | 0.347 |
| Malignant etiology (no = 0; yes = 1) | -0.037 | 0.851 |
| Interval onset of palsy to surgery (denervation time) | 0.156 | 0.427 |
| Interval surgery to evaluation | 0.112 | 0.572 |
| Classical hypoglossal-facial jump nerve suture (no = 0; yes = 1) | 0.077 | 0.572 |
| Additional upper eyelid weight (no = 0; yes = 1) | -0.025 | 0.899 |
| Sunnybrook, composite score, before facial reanimation surgery | 0.088 | 0.670 |
| Sunnybrook, composite score, after facial reanimation surgery | 0.290 | 0.134 |
| eFACE, total score, before facial reanimation surgery | -0.153 | 0.455 |
| eFACE, total score, after facial reanimation surgery | 0.189 | 0.355 |
| **FaCE Total score** |  |  |
| Gender (female = 0. male = 1) | 0.132 | 0.494 |
| Side (right = 0; left = 1) | **0.400** | **0.032** |
| Onset (Preoperative = 0; Postoperative =1) | -0.161 | 0.405 |
| Age | **-0.393** | **0.035** |
| Malignant etiology (no = 0; yes = 1) | -0.034 | 0.861 |
| Interval onset of palsy to surgery (denervation time) | -0.131 | 0.514 |
| Interval surgery to evaluation | 0.195 | 0.310 |
| Classical hypoglossal-facial jump nerve suture (no = 0; yes = 1) | 0.029 | 0.882 |
| Additional upper eyelid weight (no = 0; yes = 1) | 0.233 | 0.223 |
| Sunnybrook, composite score, before facial reanimation surgery | 0.279 | 0.158 |
| Sunnybrook, composite score, after facial reanimation surgery | **0.450** | **0.014** |
| eFACE, total score, before facial reanimation surgery | 0.020 | 0.922 |
| eFACE, total score, after facial reanimation surgery | **0.373** | **0.056** |

*p-value<0.1 in bold

**Supplementary Table 7**

| **Supplementary Table 7. Univariate analysis of associations to FDI score** | | |
| --- | --- | --- |
| **Parameter** | **rho** | **p**** |
| **FDI Physical function** |  |  |
| Gender (female = 0. male = 1) | 0.216 | 0.260 |
| Side (right = 0; left = 1) | 0.180 | 0.351 |
| Onset (Preoperative = 0; Postoperative =1) | -0.292 | 0.124 |
| Age | -0.234 | 0.223 |
| Malignant etiology (no = 0; yes = 1) | 0.034 | 0.861 |
| Interval onset of palsy to surgery (denervation time) | 0.131 | 0.497 |
| Interval surgery to evaluation | 0.169 | 0.381 |
| Classical hypoglossal-facial jump nerve suture (no = 0; yes = 1) | 0 | 1.000 |
| Additional upper eyelid weight (no = 0; yes = 1) | **0.372** | **0.047** |
| Sunnybrook, composite score, before facial reanimation surgery | 0.147 | 0.464 |
| Sunnybrook, composite score, after facial reanimation surgery | 0.176 | 0.360 |
| eFACE, total score, before facial reanimation surgery | -0.004 | 0.984 |
| eFACE, total score, after facial reanimation surgery | **0.811** | **<0.0001** |
| FDI Social/well-being function | **0.617** | **<0.0001** |
| **FDI Social/well-being function** |  |  |
| Gender (female = 0. male = 1) | 0.187 | 0.341 |
| Side (right = 0; left = 1) | 0.250 | 0.199 |
| Onset (Preoperative = 0; Postoperative =1) | -0.148 | 0.454 |
| Age | -0.253 | 0.195 |
| Malignant etiology (no = 0; yes = 1) | 0.158 | 0.422 |
| Interval between onset of palsy to surgery (denervation time) | -0.094 | 0.633 |
| Interval between surgery to evaluation | 0.278 | 0.152 |
| Classical hypoglossal-facial jump nerve suture (no = 0; yes = 1) | -0.157 | 0.425 |
| Additional upper eyelid weight (no = 0; yes = 1) | 0.110 | 0.578 |
| Sunnybrook, composite score, before facial reanimation surgery | 0.205 | 0.315 |
| Sunnybrook, composite score, after facial reanimation surgery | 0.199 | 0.310 |
| eFACE, total score, before facial reanimation surgery | 0.123 | 0.550 |
| eFACE, total score, after facial reanimation surgery | 0.184 | 0.367 |
| FDI Physical function | **0.617** | **<0.0001** |
| **FDI Total score*** |  |  |
| Gender (female = 0. male = 1) | 0.183 | 0.351 |
| Side (right = 0; left = 1) | 0.303 | 0.117 |
| Onset (Preoperative = 0; Postoperative =1) | -0.261 | 0.179 |
| Age | -0.291 | 0.134 |
| Malignant etiology (no = 0; yes = 1) | 0.150 | 0.446 |
| Interval between onset of palsy to surgery (denervation time) | 0.065 | 0.744 |
| Interval between surgery to evaluation | 0.269 | 0.167 |
| Classical hypoglossal-facial jump nerve suture (no = 0; yes = 1) | -0.172 | 0.380 |
| Additional upper eyelid weight (no = 0; yes = 1) | 0.276 | 0.155 |
| Sunnybrook, composite score, before facial reanimation surgery | 0.220 | 0.280 |
| Sunnybrook, composite score, after facial reanimation surgery | 0.196 | 0.318 |
| eFACE, total score, before facial reanimation surgery | 0.093 | 0.650 |
| eFACE, total score, after facial reanimation surgery | 0.240 | 0.238 |

*mean score of FDI Physical function and FDI Social/well-being function; **p-value<0.1 in bold

**Supplementary Table 8**

| **Supplementary Table 8. Univariate analysis of associations to the Sunnybrook composite score after* hypoglossal-facial jump nerve suture** | | |
| --- | --- | --- |
| **Parameter** | **rho** | **p**** |
| **Sunnybrook, composite score** |  |  |
| Gender (female = 0. male = 1) | -0.250 | 0.119 |
| Side (right = 0; left = 1) | 0.278 | 0.083 |
| Onset (Preoperative = 0; Postoperative =1) | -0.058 | 0.721 |
| Age | -0.109 | 0.502 |
| Malignant etiology (no = 0; yes = 1) | -0.031 | 0.848 |
| Interval between onset of palsy to surgery (denervation time) | -0.071 | 0.663 |
| Interval between surgery to evaluation | 0.209 | 0.196 |
| Classical hypoglossal-facial jump nerve suture (no = 0; yes = 1) | 0.090 | 0.581 |
| Additional upper eyelid weight (no = 0; yes = 1) | -0.040 | 0.808 |
| FaCE Facial movement | **0.518** | **0.004** |
| FaCE Facial comfort | **0.361** | **0.054** |
| FaCE Oral function | 0.311 | 0.101 |
| FaCE Eye comfort | 0.076 | 0.696 |
| FaCE Lacrimal control | 0.059 | 0.764 |
| FaCE Social function | 0.236 | 0.227 |
| FaCE Total score | **0.450** | **0.014** |
| FDI Physical function | 0.176 | 0.360 |
| FDI Social/well-being function | 0.199 | 0.310 |
| FDI Total* | 0.196 | 0.318 |

*last evaluation during follow-up; **mean score of FDI Physical function and FDI Social/well-being function; **p-value<0.1 in bold

**Supplementary Table 9**

| **Supplementary Table 9. Univariate analysis of associations to the eFACE total score after* hypoglossal-facial jump nerve suture** | | |
| --- | --- | --- |
| **Parameter** | **rho** | **p** |
| **eFACE, composite score** |  |  |
| Gender (female = 0. male = 1) | -0.076 | 0.654 |
| Side (right = 0; left = 1) | 0.162 | 0.337 |
| Onset (Preoperative = 0; Postoperative =1) | -0.021 | 0.902 |
| Age | -0.142 | 0.403 |
| Malignant etiology (no = 0; yes = 1) | 0.007 | 0.968 |
| Interval between onset of palsy to surgery (denervation time) | 0.048 | 0.778 |
| Interval between surgery to evaluation | 0.193 | 0.334 |
| Classical hypoglossal-facial jump nerve suture (no = 0; yes = 1) | 0.078 | 0.648 |
| Additional upper eyelid weight (no = 0; yes = 1) | 0.116 | 0.493 |
| FaCE Facial movement | 0.356 | 0.068 |
| FaCE Facial comfort | 0.379 | 0.051 |
| FaCE Oral function | 0.291 | 0.141 |
| FaCE Eye comfort | 0.182 | 0.364 |
| FaCE Lacrimal control | 0.108 | 0.593 |
| FaCE Social function | 0.189 | 0.355 |
| FaCE Total score | 0.373 | 0.056 |
| FDI Physical function | 0.237 | 0.233 |
| FDI Social/well-being function | 0.184 | 0.367 |
| FDI Total* | 0.240 | 0.238 |

*last evaluation during follow-up; **mean score of FDI Physical function and FDI Social/well-being function
